# Preliminary study of metabonomic changes during the progression of atherosclerosis in miniature pigs

**DOI:** 10.1002/ame2.12462

**Published:** 2024-06-25

**Authors:** Yunxiao Jia, Yuqiong Zhao, Miaomiao Niu, Changqi Zhao, Xuezhuang Li, Hua Chen

**Affiliations:** ^1^ Laboratory Animal Center Chinese PLA General Hospital Beijing People's Republic of China

**Keywords:** atherosclerosis, metabolomics, miniature pig, susceptibility

## Abstract

**Background:**

To explore potential biomarkers for early diagnosis of atherosclerosis (AS) and provide basic data for further research on AS, the characteristics of serum metabolomics during the progression of AS in mini‐pigs were observed dynamically.

**Methods:**

An AS model in Bama miniature pigs was established by a high‐cholesterol and high‐fat diet. Fasting serum samples were collected monthly for metabolomics and serum lipid detection. At the end of the treatment period, pathological analysis of the abdominal aorta and coronary artery was performed to evaluate the lesions of AS, thereby distinguishing the susceptibility of mini‐pigs to AS. The metabolomics was detected using a high‐resolution untargeted metabolomic approach. Statistical analysis was used to identify metabolites associated with AS susceptibility.

**Results:**

Based on pathological analysis, mini‐pigs were divided into two groups: a susceptible group (*n* = 3) and a non‐susceptible group (*n* = 6). A total of 1318 metabolites were identified, with significant shifting of metabolic profiles over time in both groups. Dynamic monitoring analysis highlighted 57 metabolites that exhibited an obvious trend of differential changes between two groups with the advance of time. The KEGG (Kyoto Encyclopedia of Genes and Genomes) pathway enrichment analysis indicated significant disorders in cholesterol metabolism, primary bile acid metabolism, histidine metabolism, as well as taurine and hypotaurine metabolism.

**Conclusions:**

During the progression of AS in mini‐pigs induced by high‐cholesterol/high‐fat diet, the alterations in serum metabolic profile exhibited a time‐dependent pattern, accompanied by notable disturbances in lipid metabolism, cholesterol metabolism, and amino acid metabolism. These metabolites may become potential biomarkers for early diagnosis of AS.

## Graphical abstract

An atherosclerosis model in Bama mini‐pigs was established by a high‐cholesterol/high‐fat diet, but the atherosclerotic lesions distinguished through pathological assessment of main arteries varied among individuals, which may be related to susceptibility. During the progression of atherosclerosis, the alterations in serum metabolic profile were time dependent, accompanied by notable disturbances in lipid metabolism, cholesterol metabolism, and amino acid metabolism. Those metabolites screened by monitoring dynamically may become novel biomarkers for early diagnosis of atherosclerosis.

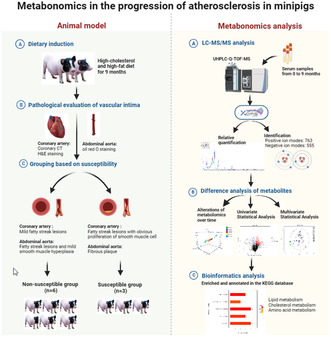

## 1 INTRODUCTION

Atherosclerosis (AS) is the primary pathological basis of coronary heart disease (CAD), ischemic stroke (IS), and peripheral vascular diseases.^1^ Among these, CAD has the highest incidence and mortality rate globally, earning the title of the “number one killer.”^2^, ^3^ CAD is a multifactorial and complex disease caused by the combined effects of genetic and environmental factors, which are closely associated with body metabolism,^4^, ^5^, ^6^ although the exact molecular mechanism remains unclear. Therefore, it is helpful to further understand the pathogenesis of AS to explore the changes in metabolomics during the progression of AS.

The severity of CAD in human patients is disturbed by a variety of factors, whereas animal models can minimize the impact of nonpathogenic factors. At present, genetically modified mouse models are the most commonly used for AS research, followed by the domestic rabbits, whereas nonhuman primates and pigs are considered to be more valuable models for translational medicine.^7^, ^8^ There are similarities between mini‐pigs and humans in terms of cardiovascular anatomy, lipid profile, metabolic mechanism, pathophysiology, and diet structure, especially the progression and pathological tissue morphology of AS plaques, which are more similar than rodent models, with high reproductive rate and ethical acceptability.^9^, ^10^, ^11^ Mini‐pigs have emerged as the most suitable animal for studying the mechanism of metabolic diseases such as AS, and its application in the research of cardiovascular diseases and metabolic disorders has shown a significant growth trend in recent years.^10^, ^11^, ^12^, ^13^ Therefore, we chose mini‐pigs to carry out animal experimental research, which is convenient to observe the progression of AS dynamically and accurately determine the development process and severity of AS through pathological research.

Up to now, the clinical diagnosis of CAD mainly relies on imaging techniques or early evaluation through the identification of biomarkers for high‐risk factors of AS.^14^, ^15^ However, these traditional diagnostic methods have certain limitations.^16^, ^17^ Metabolites reveal the dynamic process of cellular homeostasis. Metabolomics can comprehensively characterize the flux of small‐molecule metabolites such as amino acids, lipids, organic acids, and their relationship with biological phenotype, which can more directly and accurately reflect the physiological state of the organism, providing more valuable insights for diagnosis, prognosis, and pathogenesis research of disease. Metabolomics has been widely applied in the research of human AS, as well as the risk assessment and prediction of CAD.^18^, ^19^, ^20^, ^21^ Early diagnosis of AS is essential to prevent atherosclerotic plaque rupture and thrombosis, which can effectively intervene for patients and reduce the mortality and complications associated with cardiovascular diseases. Therefore, finding new and faster diagnostic methods, as well as new therapeutic targets, is crucial for the early treatment of AS.

In this study, the AS model in mini‐pigs was established inducing by a high‐cholesterol/high‐fat diet. Susceptible and non‐susceptible animals were determined based on pathological detection. Serum metabolomics changes were dynamically monitored using ultra‐high performance liquid chromatography tandem mass spectrometry (UHPLC‐MS/MS). Subsequently, a comparative analysis between susceptible and non‐susceptible animals was performed to investigate the relationship between changes in serum metabolic and the progression of AS in miniature pigs.

## 2 METHODS

### 2.1 Animal experiments

Nine male Bama mini‐pigs, 6 months old and 12–15 kg weight, were purchased from the Beijing Shi Chuang Century Mini‐pig Breeding Base (Beijing, China). During the experiment, the animals were housed in a stable environment at a temperature of 20°–26°C, 40%–70% relative humidity, and a 12‐h light/dark cycle. The animals were fed in single cages and allowed to drink freely.

All animals were initially fed a standard diet for 1 week to adapt to the environment. Subsequently, they were all switched to a high‐cholesterol/high‐fat diet consisting of 82.5% standard diet, 15% tallow, 2% cholesterol, and 0.5% bile salt, which was continued for 9 months. The daily feeding amount was given at 3% of body weight and adjusted based on monthly changes in body weight. All feed was purchased from Beijing Keao Xieli Feed Co., Ltd. (Beijing, China). Fasting serum samples were collected from veins once a month, a portion of which was used for detection of blood lipid indexes, and the remaining portion was stored in the refrigerator at −80°C until metabolomics analysis.

### 2.2 Serum lipid level monitoring

The serum lipid indexes, including triglyceride (TG), total cholesterol (TC), high‐density lipoprotein (HDL‐C), low‐density lipoprotein (LDL‐C), and free fatty acid (FFA), were measured once a month. The AS index (AI) was calculated, AI = (TC‐HDL‐C)/HDL‐C. All assays were performed by Beijing North Institute of Biotechnology Co., Ltd. (Beijing, China) with a Toshiba 120 automatic biochemical instrument. The reagents were provided by Beijing Bei Jian Xin Chuang Yuan Biotechnology Co., Ltd. (Beijing, China).

### 2.3 Coronary computed tomography angiography

At the ninth month of treatment, animals were anesthetized using xylazine hydrochloride (15–25 mg/kg) and midazolam (0.15–0.25 mg/kg) via intramuscular injection, followed by maintenance of anesthesia with intravenous injection of 3% pentobarbital sodium (3 mg/kg). Cardiac coronary arteries were examined with a Siemens SOMATOM Definition Flash dual‐source computed tomography (CT) in the left lateral decubitus position. The test‐bolus technique was adopted to estimate the dosage of contrast agent. Then, 15 mL iodophor (350 mg/mL) was injected through the auricular vein at a flow rate of 3.0 mL/s using a double‐barrel high‐pressure syringe. A layer was chosen at the level of the aortic root, and the time‐density curve was measured by an automatic software to establish the scanning delay time, and then the coronary artery scan was performed from 1 cm below the carina of tracheal to 2 cm below the diaphragm. A volume of 50–60 mL contrast agent was injected at a flow rate of 3 mL/s, followed by 30 mL of normal saline at the same flow rate. The scanning layer thickness was 0.6 mm, with a rotating speed of 0.28 s/360°, tube voltage of 120 kV, and tube current of 222 mA. A prospective electrocardiograph (ECG) gated scanning mode was adopted, with an acquisition time window of 48%–72% of the cardiac cycle and a scanning duration of approximately 8 s.

### 2.4 Pathological evaluation of vascular intima

At the end of the 9‐month experiment, the animals were euthanized via exsanguination from the femoral artery while under anesthesia and immediately underwent dissection. Following Stary criteria,^22^, ^23^, ^24^ the pathological diagnosis of AS lesions was performed. The abdominal aorta and coronary artery were dissected carefully. The abdominal aorta was opened longitudinally and immersed in a saturated oil red O staining solution for 6 min. Subsequently, the tissues were photographed and fixed in 10% formalin solution. To measure the proportion of atherosclerotic plaque in the intima of the abdominal aorta, the photographs were cut between the renal artery branch at the cephalic end and the iliac artery branch at the caudal end. Then, the scope of the plaque lesions was outlined meticulously, and the proportion of the plaque lesion area to the intimal area of the abdominal aorta was calculated using an Image‐Pro Plus 4.5 software. The anterior descending branch of the left coronary artery, along with myocardium, was collected and fixed in 10% formalin solution. For histopathologic examination of the abdominal aorta and coronary artery, the most prominent parts of the plaque bulge lesions were selected for paraffin‐embedding, sectioning, and hematoxylin and eosin (H&E) staining, and observed under an optical microscope (OLYMPUS BX51, Tokyo, Japan).

### 2.5 Serum metabolite extraction

The serum samples from nine mini‐pigs stored at −80°C for 0–9 months were transported on dry ice to Shanghai Applied Protein Technology Co., Ltd. for detection. After being transported, the samples were thawed slowly at 4°C and mixed with an equal volume of cold extraction solvent composed of methanol/acetonitrile/H_2_O (2:2:1, v/v/v). After adequately vortexed, the samples were sonicated at low temperature for 30 min and then incubated at −20°C for 10 min. Next, the samples were centrifuged at 14000 *g* for 20 min at 4°C. The resulting supernatants were collected and lyophilized in a vacuum centrifuge maintained at 4°C to prevent degradation. For LC–MS/MS analysis, the lyophilized samples were redissolved in 100 μL of a solvent mixture of acetonitrile/H_2_O (1:1, v/v) and transferred to LC vials.

### 2.6 LC–MS/MS analysis

Referencing a published untargeted metabolomics protocol,^25^ the high‐resolution untargeted metabolomic assay was performed using an UHPLC system (1290 Infinity LC, Agilent Technologies, Santa Clara, CA, USA) coupled with a quadrupole time‐of‐flight mass spectrometer (AB SCIEX Triple TOF 6600) interfacing with an electrospray ionization (ESI) source.

Chromatographic separation was performed on Hydrop Interaction Liquid Chromatography (HILIC) column (2.1 × 100 mm, 1.7 μm particle size, Waters, Ireland) with a column temperature of 25°C and flow rate of 0.5 mL/min. A 2‐μL aliquot from LC vials was injected into an automatic sampler at 4°C and analyzed continuously in random order to mitigate the impact of instrument detection signal fluctuations. The gradient elution was achieved using solvent A (25 mmol/L ammonium acetate and 25 mmol/L ammonia in water) and solvent B (acetonitrile), following a previously established elution procedure.^26^ The mass spectrometer was operated in both positive and negative ionization modes using ESI. The primary and secondary spectra of the samples were collected. The experimental procedure and data acquisition for analysis were carried out according to a previous study.^27^

### 2.7 Data processing and analysis

The raw MS data (Wiff. scan files) were converted into MzXML files using ProteoWizard MSConvert before being imported into freely available XCMS software for peak alignment and area extraction. Compound identification of metabolites was performed by comparing the accuracy of *m/z* value (<10 ppm) and MS/MS spectra with an in‐house database established using available authentic standards (Shanghai Applied Protein Technology Co., Ltd., Shanghai, China).^28^ The identification results were strictly manually verified and confirmed.

After sum‐normalization, the processed data were analyzed by R package. When comparing two groups, *t*‐test and fold change (FC) analysis were applied to evaluate the differential metabolites. *p*‐values resulting from the *t*‐test indicate the significance level of metabolite difference between groups, and FC values describe the increase or decrease folds of mean value between compared groups. The metabolites with *p‐*values < 0.05 and FC > 1.5 or <0.67 were considered significantly different. The multivariate data analysis was performed in SIMCA‐P software (version 14.1, Umetrics, Umea, Sweden), including Pareto‐scaled principal component analysis (PCA) and partial least squares discriminant analysis (PLS‐DA).

The significant differential metabolites from 0 to 9 months were individually screened and merged, and duplicates were removed. Due to the impact of anesthetization on the organism metabolism, the data from the ninth month (i.e., for animals that underwent anesthesia) were excluded from the dynamic analysis. The dynamic changes in differential metabolites in every month were analyzed, and metabolites with significant trends were selected for bioinformatics analysis. The KEGG (Kyoto Encyclopedia of Genes and Genomes) database (http://www.kegg.jp/) was utilized for annotating and enriching the corresponding metabolic pathways. To assess the impact of metabolites, the significance levels of metabolic pathways were analyzed using Fisher's exact test. Pathways with *p*‐values < 0.05 were considered significantly associated with metabolic or signal transduction pathways.

### 2.8 Statistical analyses

Except those related to metabolomics, the statistical analyses of all experimental data were performed with SPSS Statistics (version 26.0, Chicago, IL, USA) and GraphPad Software (version 8.0, SanDiego, CA, USA). Data were presented as mean ± standard error of the mean (SEM). For group comparison analysis, an independent‐sample *t*‐test was carried out with SPSS. The *p*‐value < 0.05 (two‐tailed) was considered statistically significant.

## 3 RESULTS

### 3.1 Atherosclerotic lesion analysis

Coronary CT examination showed that the coronary artery intima of all animals was unobstructed without obvious lumen stenosis. Among them, endothelial roughness was observed in the intima of the anterior descending branch of the left coronary artery in three animals, indicating mild plaque lesions (data not shown).

**FIGURE 1:**
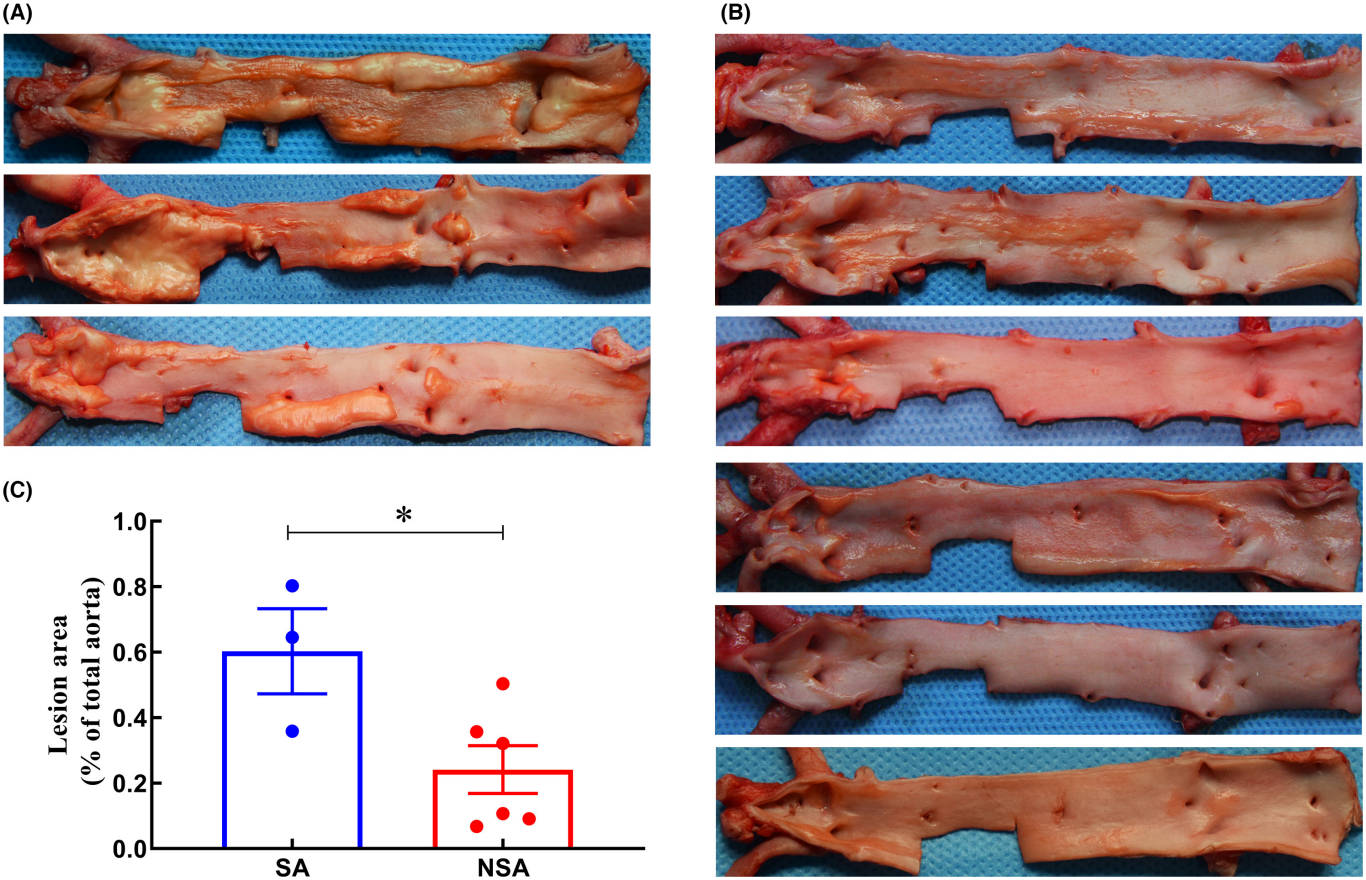
Area of intimal lesions in the abdominal aorta. The proportion of the area of atherosclerotic plaque lesion to the intima in abdominal aorta between the renal artery and the iliac artery branch stained with oil red O solution were measured. (A) The atherosclerosis susceptible (SA) group (*n* = 3). (B) The atherosclerosis non‐susceptible (NSA) group (*n* = 6). (C) The lesion area was significantly increased in the SA group compared to the NSA group. *p < 0.05.

**FIGURE 2:**
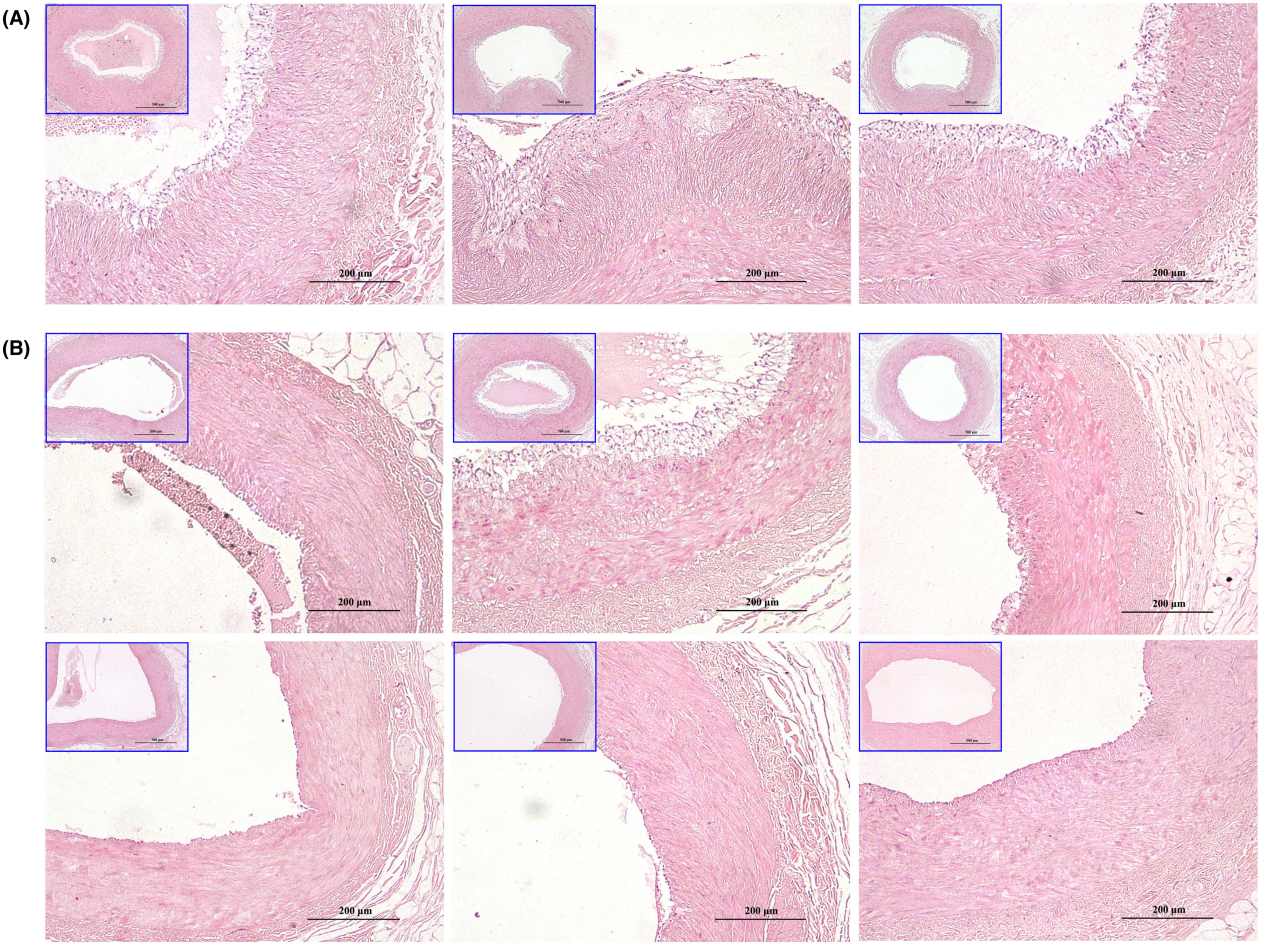
Histopathologic examination of coronary artery cross‐sections (100× and 200×). The base of the anterior descending branch of the left coronary artery was stained with hematoxylin and eosin (H&E). (A) In the atherosclerosis susceptible (SA) group (*n* = 3), visible proliferation of intimal smooth muscle cells along with an obvious aggregation of foam cell was observed in the intima of coronary arteries. (B) In the atherosclerosis non‐susceptible (NSA) group (*n* = 6), noticeable intimal lesions with varying deposition of foam cell were observed in two cases without evident smooth muscle cell proliferation yet. Nevertheless, mild intimal lesions with sporadic observation of foam cells and no thickening of the vascular intima in the other four cases.

As shown in Figure 1, fatty streak lesions or plaque lesions were observed within the intima of the abdominal aorta in all animals. Among them, three animals exhibited the most severe plaque lesions with serious prominent bulge, accounting for 35.91%, 64.57%, and 80.30% of the total area of intima, respectively (Figure 1A). Meanwhile, pathological examination of the abdominal aorta (Figure S1A) revealed that the lesions were mainly fibrous plaque composed of numerous proliferating smooth muscle cells along with infiltrating foam cells and inflammatory cells. In some cases, nourishing blood vessels passing through the middle membrane into plaques or necrotic nuclei were observed, although not extensively. Notably, obvious lesions characterized by visible proliferation of intimal smooth muscle cells were observed in the corresponding coronary arteries of these three animals, as shown in Figure 2A. Consequently, these three animals were classified as AS susceptible group (SA).

The lesions observed in the abdominal aorta of the remaining six animals were slight, with the proportion of plaque lesions in vascular intima ranging from 6.79% to 50.34% (Figure 1B). These lesions mainly consisted of fatty streaks and mild smooth muscle hyperplasia, without obvious fibrous plaques observed under the microscope (Figure S1B). The pathological examination of corresponding coronary artery in these six animals, presented in Figure 2B, revealed that only two out of the six animals displayed noticeable intimal lesions characterized by varying degrees of foam cell deposition and without evident smooth muscle cell proliferation. The other four animals also exhibited mild intimal lesions without thickening of the vascular intima and sporadic observation of foam cells. As a result, these animals were categorized as AS non‐susceptible group (NSA). The statistical results (Figure 1C) demonstrated a significant difference in the proportion of plaque lesion area between SA and NSA groups (*p* < 0.05).

### 3.2 Analysis of serum lipids

**FIGURE 3:**
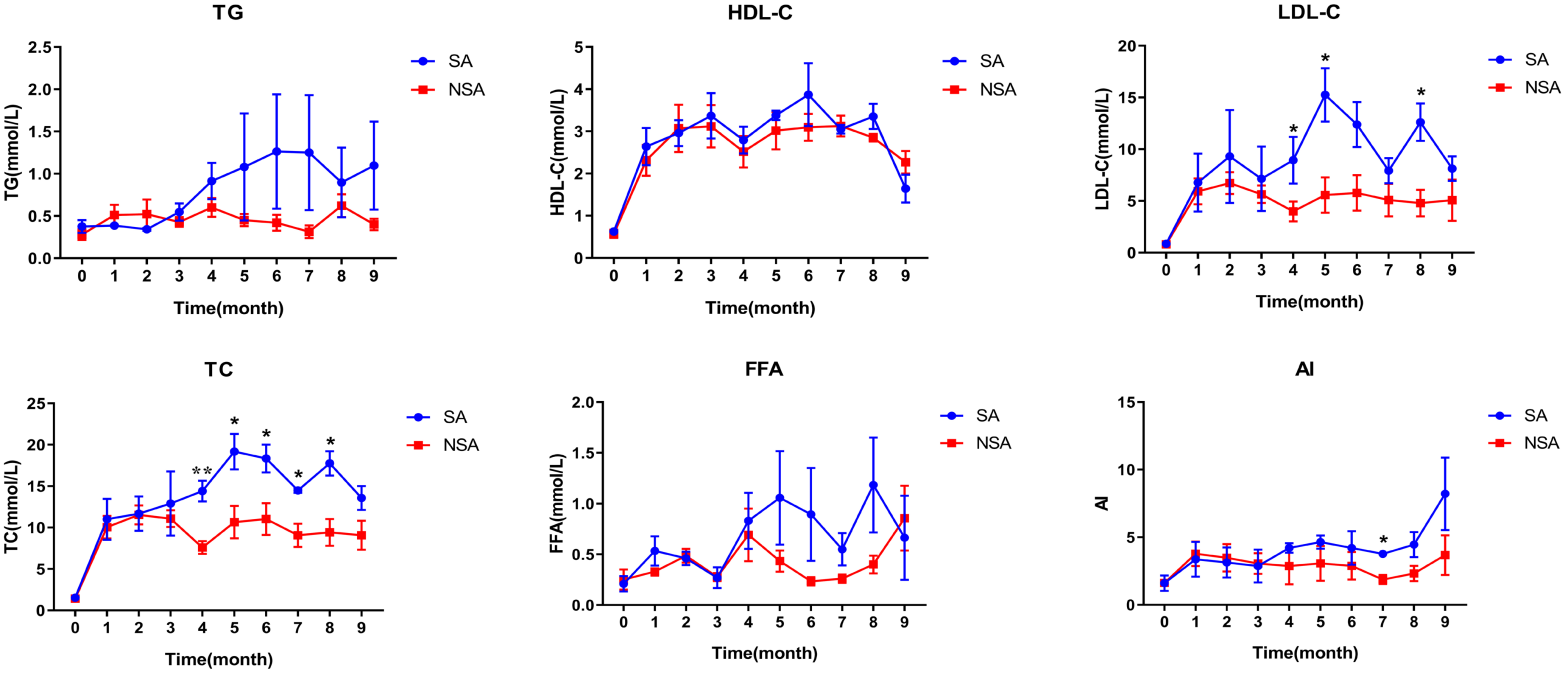
Dynamic monitoring of serum lipid indexes during the experiment (*p < 0.05; **p < 0.01). AI, atherosclerosis index; FFA, free fatty acid; HDL‐C, high‐density lipoprotein; LDL‐C, low‐density lipoprotein; TC, total cholesterol; TG, triglyceride.

The results of lipid index monitoring are presented in Figure 3. Prior to the experiment, the levels of lipid indexes among nine animals were generally consistent. Following the consumption of a high‐cholesterol/high‐fat diet, there was an increase in variation in the serum TG and FFA levels among individuals in both groups starting from the fourth month, although no significant difference was observed in the average values. Notably, the levels of TC, LDL‐C, and HDL‐C significantly increased as a result of the dietary intervention. Although there was no notable disparity in the levels of HDL‐C between the two groups, the levels of TC and LDL‐C in the SA group showed a trend of increase compared to the NSA group, with certain time points showing significant differences (TC, at the fourth to eighth month, *p* < 0.05; LDL‐C, at the fourth, fifth, and eighth month, *p* < 0.05).

Prior to the experiment, the average values of AI in SA and NSA groups were 1.6 and 1.62, respectively. During the high‐cholesterol/high‐fat diet intervention period, the AI values in the NSA group maintained slight fluctuation, with average values ranging from 1.87 to 3.78. Conversely, the average values of AI in the SA group fluctuated from 2.87 to 8.22, slightly higher than those in the NSA group, especially during 4–9 months (only significant at the seventh month, *p* < 0.05).

### 3.3 Global metabolic profiling

The global metabolic profiles of serum from all animals were acquired using LC–MS/MS analysis. A total of 1318 metabolites were identified, including 763 and 555 metabolites in positive and negative ion modes, respectively.

**FIGURE 4:**
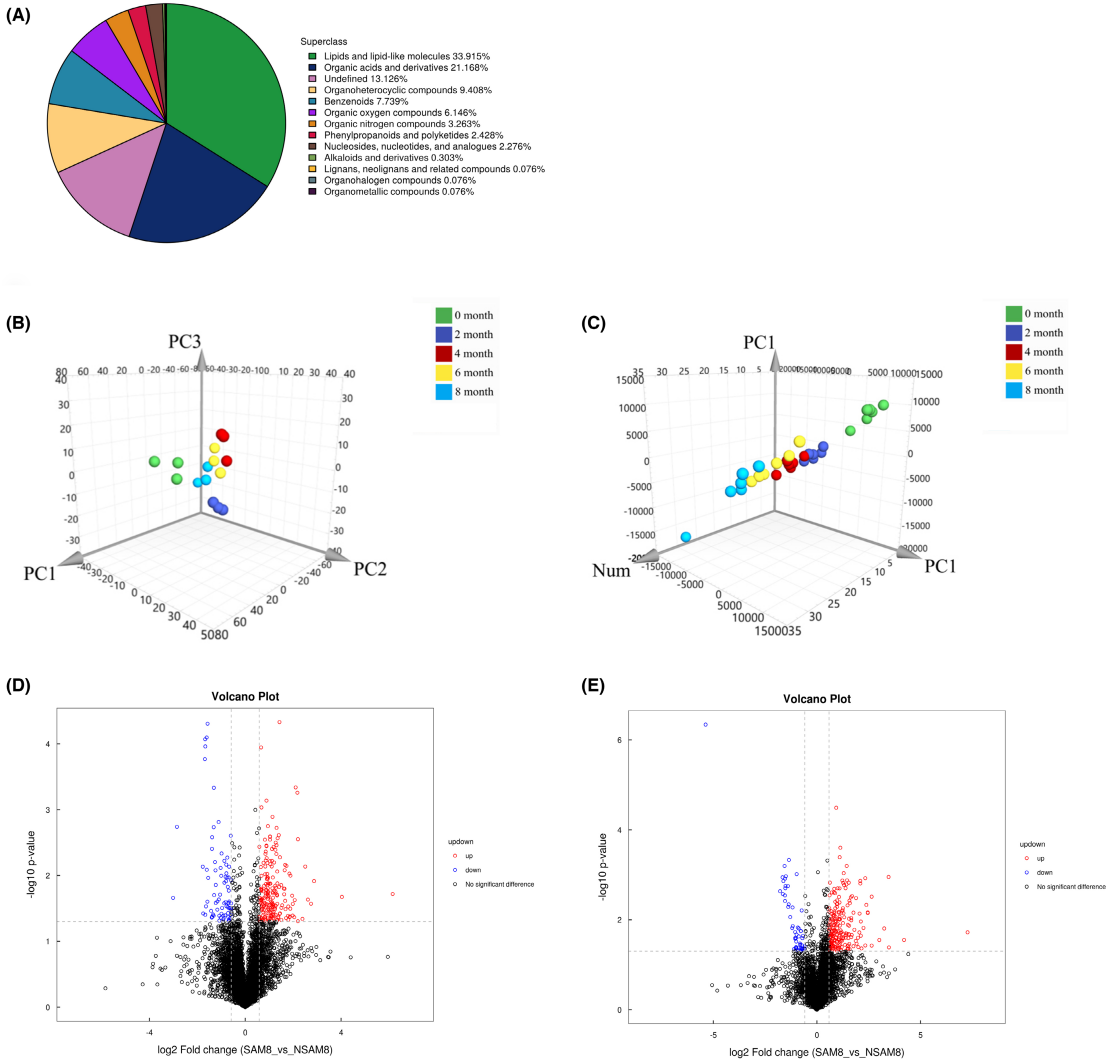
The global metabolic profiling and variation analysis of serum metabolic. (A) The proportion of metabolites in each chemical classification. (B) The 3D score maps generated for partial least squares discriminant analysis (PLS‐DA) analysis of metabolism profile in atherosclerosis susceptible (SA) group, showing three principal components with R^2^X = 0.436, R^2^Y = 0.637, and Q^2^ = 0.103. (C) The 3D score maps generated for PLS‐DA analysis of metabolism profile in atherosclerosis non‐susceptible (NSA) group, with one principal component and R^2^X = 0.321, R^2^Y = 0.167, and Q^2^ = 0.123. The volcanic maps of fold change (FC) analysis in negative ion modes (D) and positive ion modes (E) between SA and NSA groups at the eighth month. Red represents differential metabolites with FC>1.5 and *p*<0.05, whereas blue represents differential metabolites with FC < 0.67 and *p* < 0.05.

All the identified metabolites were categorized based on their chemical taxonomy attribution information (Figure 4A). Among them, lipids and lipid‐like molecules were the most abundant accounting for 33.915%, followed by organic acids and derivatives (21.168%), organic heterocyclic compounds (9.408%), benzene compounds (7.739%), organic oxygen compounds (6.146%), organic nitrogen compounds (3.263%), phenylpropane and polyketide compounds (2.428%), and nucleotides and analogues (2.276%). Additionally, alkaloids and derivatives accounted for a minor proportion at 0.303%, whereas lignans, neolignane and related compounds, organic halogen compounds, and organic metal represented only a small fraction at 0.076%. So far, a portion of the identified metabolites (13.126%) remains unclassified.

### 3.4 Alterations in metabolomics after feeding high‐cholesterol/high‐fat diet

The PLS‐DA analysis was performed on the metabolites at 0, 2, 4, 6, and 8 months. The changes in metabolic profiles at various time points within the SA and NSA groups could be distinguished effectively using the PLS‐DA model. The three‐dimensional scoring map of PLS‐DA model (Figure 4B,C) illustrated that the metabolic profiles in both SA and NSA groups shifted significantly over time. The distance between data points at 2 and 0 months were notably far, indicating that the high‐cholesterol/high‐fat diet had greatly disturbed the metabolomics of animals. During the progression of hypercholesterolemia to early AS, the metabolomics of mini‐pigs underwent significant shifts.

### 3.5 Variation analysis between SA and NSA

FC analysis and *t*‐test were performed for all metabolites identified in both positive and negative ion modes. To assess the significance of metabolites between the SA and NSA groups, metabolites with FC > 1.5 or FC < 0.67 and *p*‐value < 0.05 were visually represented in volcanic maps. The volcano plots for the eighth month are displayed in Figure 4D,E as representative results, whereas the additional results can be acquired in Figure S2.

The overall distribution trend and degree of variation in samples within or between groups were observed by PCA analysis. The PCA analysis of each month showed the R^2^ values >0.5, indicating a reliable model with significant differences between two groups and noticeable individual differences within groups. Metabolites with qualitative names and FC > 1.5 or FC < 0.67, along with *p‐*value < 0.05, were considered as the criteria for screening significantly differential metabolites. Statistical analysis revealed that there were significant differences in metabolites between the SA and NSA groups in each month. Initially, the number of differential metabolites was handful between the two groups in the first 3 months (9–36 kinds), whereas they gradually increased (70–163 kinds) from the fourth month onward. Some metabolites showed significant differences only in specific individual months and were subsequently eliminated for further analysis (data not shown).

**FIGURE 5:**
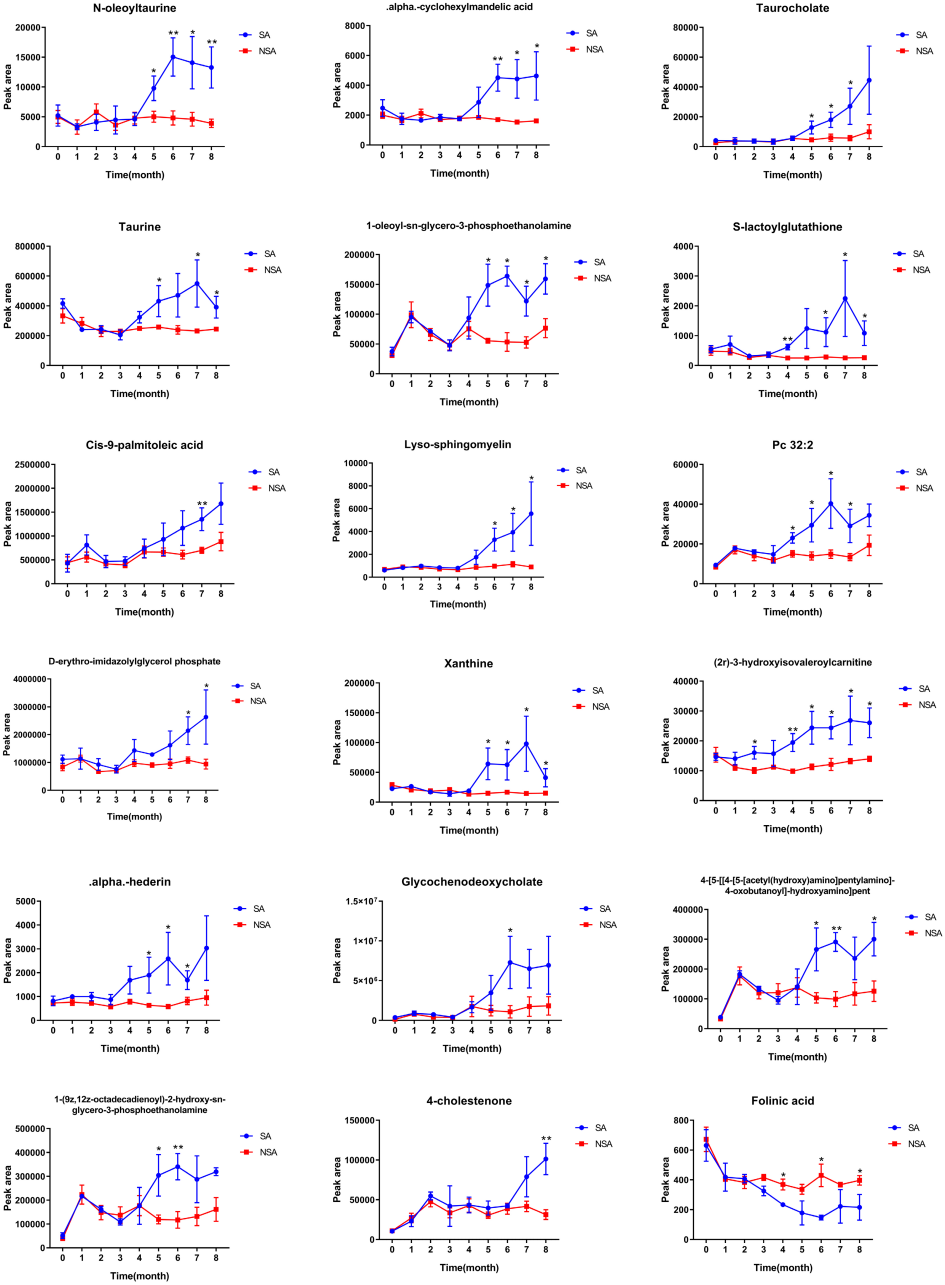
Representative results of the dynamic analysis of 57 metabolites during 0–8 months (*p < 0.05; **p < 0.01).

To explore potential metabolites as biomarkers in the development process of AS, all differential metabolites from 0 to 9 months were merged and de‐weighted. Subsequently, the dynamic changes in these differential metabolites were analyzed across months. The results of the dynamic analysis, as shown in Figure 5 and Figure S3, revealed that 57 metabolites were found relatively significant trends of variation between the SA and NSA groups, including 50 metabolites upregulated and 7 metabolites downregulated. In the NSA group, the levels of these metabolites remained generally consistent compared to 0 month throughout the experiment, with slight fluctuations but no obvious changes. During the first 4 months of experiment, the metabolite levels in the SA group were essentially consistent with those in the NSA group, indicating no significant differences (*p* > 0.05) despite the high‐cholesterol/high‐fat diet. However, partial metabolites in the SA group showed gradually ascending or declining trends compared to the NSA group from the fourth month onward, most of which showed statistical significance within 2 to 4 months (*p* < 0.05), and a few metabolites showed significant differences until the seventh or eighth month (*p* < 0.05).

### 3.6 Metabolic pathway disorder

**FIGURE 6:**
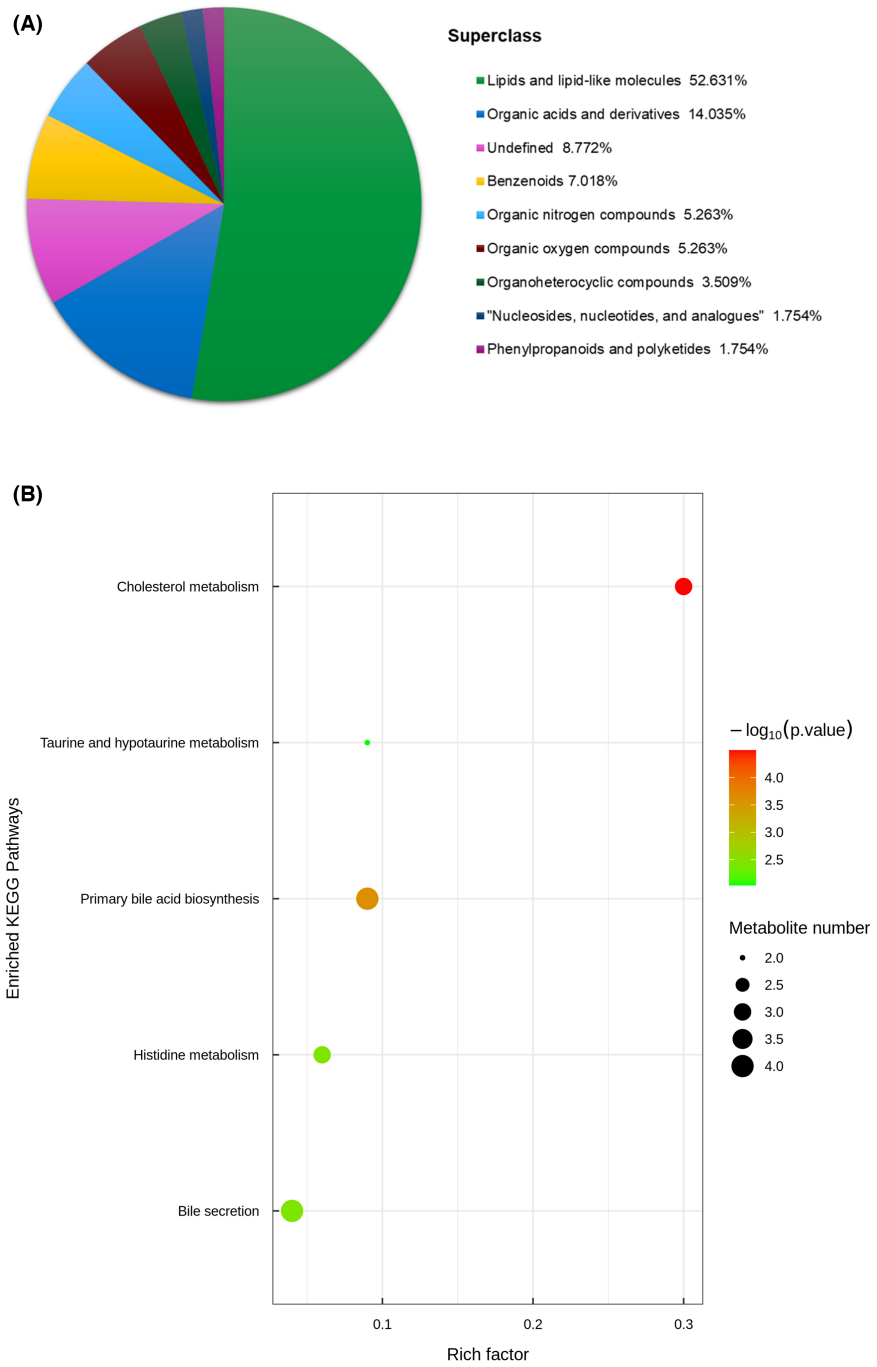
Bioinformatics analysis of differential metabolites. (A) Statistical analysis of the chemical classification of 57 metabolites. (B) The bubble diagram of metabolic pathway enriched in KEGG database. Each bubble in the diagram represents a metabolic pathway, with the abscissa and size of the bubble indicating the impact factor of the pathway in topology analysis. Larger bubbles correspond to pathways with greater impact. The vertical axis and color of the bubble reflect the *p*‐value of enrichment analysis (taken as −log10 *p*‐value). Darker colors indicate smaller *p*‐values and more significant enrichment. The rich factor denotes the proportion of differential metabolites in the pathway compared to the total number of metabolites annotated in the pathway.

The chemical classification of the above metabolites was determined through statistical analysis, as shown in Figure 6A. The 57 metabolites could be roughly categorized into eight classes, among which lipids and lipid‐like molecules were the most abundant (52.631%), followed by organic acids and their derivatives (14.035%), benzenes (7.018%), organic nitrogen compounds (5.263%), organic oxygen compounds (5.263%), organic heterocyclic compounds (3.509%), nucleotides and analogues (1.754%), phenylpropane and polyketide compounds (1.754%), and the metabolites unclassified at present account for 8.772%.

Through the KEGG database, a total of 28 metabolites were successfully annotated as detailed in Table 1, among which 18 metabolites were enriched into the metabolic pathway and 10 metabolites were not enriched. These annotated metabolites play key roles in various metabolic processes, such as lipid metabolism, amino acid metabolism, digestive system metabolism, fatty acid metabolism, nucleotide metabolism, energy metabolism, as well as signal transduction pathways.

**TABLE 1:** The annotation results of metabolites in the KEGG database.

Metabolite	KEGG ID	Map name	Pathway hierarchy
l‐Palmitoylcarnitine	C02990	Fatty acid degradation	Lipid metabolism
		Fatty acid metabolism	
d‐Erythro‐imidazolylglycerol phosphate	C04666	Histidine metabolism	Amino acid metabolism
		Biosynthesis of amino acids	
1‐Methylhistamine	C05127	Histidine metabolism	Amino acid metabolism
4‐Imidazoleacetic acid	C02835	Histidine metabolism	Amino acid metabolism
Folinic acid	C03479	One carbon pool by folate	Metabolism of cofactors and vitamins
*N*‐Oleoylethanolamine	C20792	cAMP signaling pathway	Signal transduction
S‐Lactoylglutathione	C03451	Pyruvate metabolism	Carbohydrate metabolism
Zymosterol	C05437	Steroid biosynthesis	Lipid metabolism
Taurine	C00245	Primary bile acid biosynthesis	Lipid metabolism
		Taurine and hypotaurine metabolism	Metabolism of other amino acids
		Sulfur metabolism	Energy metabolism
		ABC transporters	Membrane transport
		Neuroactive ligand‐receptor interaction	Signaling molecules and interaction
Cis‐9‐palmitoleic acid	C08362	Fatty acid biosynthesis	Lipid metabolism
Glycochenodeoxycholate	C05466	Primary bile acid biosynthesis	Lipid metabolism
		Bile secretion	Digestive system
		Cholesterol metabolism	
d‐(+)‐Melibiose	C05402	Galactose metabolism	Carbohydrate metabolism
		ABC transporters	Membrane transport
Lovastatin hydroxy acid	C21130	Metabolic pathways	Global and overview maps
Taurochenodeoxycholate	C05465	Primary bile acid biosynthesis	Lipid metabolism
		Bile secretion	Digestive system
		Cholesterol metabolism	
Taurocholate	C05122	Primary bile acid biosynthesis	Lipid metabolism
		Taurine and hypotaurine metabolism	Metabolism of other amino acids
		Cholesterol metabolism	Digestive system
		Bile secretion	
Taurolithocholic acid sulfate	C03642	Bile secretion	Digestive system
Thymidine	C00214	Pyrimidine metabolism	Nucleotide metabolism
Xanthine	C00385	Purine metabolism	Nucleotide metabolism
		Caffeine metabolism	Biosynthesis of other secondary metabolites
4‐Cholestenone	C00599		
Azlocillin	C06839		
Glycolithocholic acid	C15557		
Irbesartan	C07469		
Alpha.‐hederin	C08954		
1 h‐imidazo[4,5‐c]pyridine‐6‐carboxylicacid,1‐[[4‐(dimethylamino)‐3‐methylphenyl]methyl]‐5‐(2,2‐diphenylacetyl)‐4,5,6,7‐tetrahydro‐, (6 s)‐	C15553		
Glycodeoxycholic acid	C05464		
Lithocholylglycine	C15557		
Maltotetraose	C02052		
Swertiamarin	C09800		

*Note*: Metabolite represents the name of the metabolite with significant differences; KEGG ID represents the unique KEGG ID number of the metabolite; map name indicates the name of the metabolic pathway; pathway hierarchy illustrates the classification and attribution of metabolic pathways.

Abbreviation: KEGG, Kyoto Encyclopedia of Genes and Genomes.

Several significantly impacted metabolic and signal transduction pathways were highlighted using the KEGG database, as illustrated in Figure 6B. Among these pathways, five metabolic pathways were enriched with significant differences (*p* < 0.05), namely cholesterol metabolism, primary bile acid biosynthesis, histidine metabolism, taurine and hypotaurine metabolism, as well as bile secretion. Particularly noteworthy were the cholesterol metabolism (*p* < 0.01) and primary bile acid biosynthesis (*p* < 0.01) pathways, which exhibited even greater significance compared to others. The metabolites enriched in these significant metabolic pathways mainly include taurocholate, taurine, d‐erythro‐imidazolylglycerol phosphate, 1‐methylhistamine, 4‐imidazoleacetic acid, glycochenodeoxycholate, taurochenodeoxycholate, and taurolithocholic acid sulfate.

## 4 DISCUSSION

CAD is a complex disease with genetic susceptibility, where the risk of developing CAD in individuals is modulated by the interaction of genetic factors and lifestyle. Studies have demonstrated a consistent risk of CAD among close relatives, with genetic factors accounting for approximately 50% of total fatal CAD cases.^29^, ^30^ The genetic contribution rate of CAD is estimated to be between 40% and 50%.^31^ CAD originates in the early stages of life, with a slow accumulation of lesions over decades before clinical symptoms such as myocardial infarction and sudden cardiac death manifest. Preventive measures initiated in the early life are crucial to avoid such clinical events. Clinically, the onset of CAD in patients can be complex due to multiple factors, whereas animal experiments can provide convenient means for exploring early diagnostic indicators.

In this experiment, the AS model in Bama mini‐pigs was established with a high‐cholesterol/high‐fat diet over 9 months, yielding consistent results as previously reported in our preliminary studies.^32^ The vascular lesions in animals were accurately assessed through pathological examination of abdominal aorta and coronary arteries, revealing varying lesion characteristics among individuals. Based on the severity of lesions, mini‐pigs were divided into SA (*n* = 3) and NSA groups (*n* = 6). At the same time, the difference in susceptibility was also further evidenced in blood lipid indicators (TC and LDL‐C). Due to consistent experimental conditions, the susceptibility disparity primarily reflects the impact of genetic factors in animals.

The occurrence and development of AS is a complex and gradual process. Several studies on animal models have revealed significant changes in metabolomics during the progression from hypercholesterolemia to early AS, among which cholesterol metabolism, fatty acid metabolism, amino acid metabolism, and phospholipid metabolism play an important role in the progression of AS.^33^, ^34^, ^35^, ^36^, ^37^ Similarly, in our research, the metabolic profiles of both SA and NSA groups evolved over time, with gradual differences in metabolite levels between the two groups. This indicated that the metabolic changes were time dependent during the progression of AS induced by high‐cholesterol and high‐fat diet in mini‐pigs.

In this study, 57 metabolites were analyzed with a focus on lipids and lipid‐like molecules due to their significant alterations, indicating a major role of lipid metabolism in the progression of AS. The signal pathway analysis enriched in the KEGG database revealed that cholesterol metabolism, primary bile acid biosynthesis, histidine metabolism, as well as taurine and hypotaurine metabolism were significantly impacted in the progression of AS induced by high‐cholesterol/high‐fat diet, with the cholesterol metabolism and primary bile acid biosynthesis pathways being particularly noteworthy. Previous studies have proved consistent with our findings.35 Furthermore, there is evidence that certain drugs impact the progression of AS mainly by interfering with cholesterol metabolism or primary bile acid biosynthesis pathways.^38^, ^39^, ^40^, ^41^, ^42^ Dynamically monitoring the changes in metabolites during the progression of AS, we found that the levels of metabolites such as glycochenodeoxycholate, taurochenodeoxycholate, and taurocholate in lipids increased gradually in the SA group from the fourth month of experiment, significantly surpassing levels in the NSA group and participating in cholesterol metabolism and primary bile acid biosynthesis. These changes basically align with the dynamic changes in blood lipids in animals, indicating a potential link between metabolites and the susceptibility to AS. Additionally, taurine, classified as an organic acid, also plays a role in the primary bile acid biosynthesis pathway.

A substantial body of evidence from rodent models and cohort studies has shown that amino acid metabolism plays a significant role in the development of AS.^43^, ^44^, ^45^, ^46^, ^47^ Our findings align with this, demonstrating alterations in histidine metabolism, taurine and hypotaurine metabolism pathways. Recent research on acute ischemic stroke has further highlighted changes in fatty acid and amino acid metabolism in plasma among stroke patients with different subtypes, including arginine, proline, and histidine metabolism.^48^ Despite limited reports on the role of histidine metabolism in diseases related to AS, our research revealed significant differences in key metabolites such as 4‐imidazoleacetic acid, d‐erythro‐imidazolylglycerol phosphate, and 1‐methylhistidine involving in histidine metabolism between groups induced by a high‐cholesterol/high‐fat diet, which is a new discovery worthy of attention. Notably, taurine and taurocholate, known for their involvement in various metabolic reactions and diseases, have been proven to be related to AS in multiple studies.^49^, ^50^ Similarly, our experiment results indicated that the exacerbation of AS lesions correlates with upregulated levels of these metabolites in serum.

Numerous metabonomic studies on AS have consistently demonstrated significant disruptions in fatty acid metabolism. For instance, Xi Chen et al.^51^ analyzed the plasma metabolomics from 516 patients of AS and 528 healthy individuals using GC/MS in 2010, and indicated a direct association between disrupted fatty acid metabolism, especially palmitate metabolism, which was confirmed as a phenotypic biomarker for clinical diagnosis of AS. In contrast, there were no significant alterations in the fatty acid metabolism pathway between the susceptible and non‐susceptible animals in our research, possibly owing to the limited samples size. Nonetheless, the dynamic analysis revealed a gradual increase in l‐palmitoylcarnitine and cis‐9‐palmitoleicacid levels involved in fatty acid biosynthesis and degradation in the SA group compared to the NSA group after the third month of induction with high‐cholesterol/high‐fat diet. This suggested a progressive disruption in fatty acid metabolism pathways.

In addition, other metabolites that showed differences in dynamic analysis may be related to the occurrence and development of AS. Although no significant changes in metabolic pathways were enriched or reported to be related to AS, these metabolites are worthy of further research and discussion. For example, S‐lactoylglutathione involved in pyruvate metabolism and many enzymatic reactions can be a potential marker for metabolic disorders such as diabetes or ketosis, and also be converted into glutathione, which is involved in glutathione biosynthesis pathway and has been investigated for its role in the progression of AS by scholars.^52^ Trimethylamine can be further metabolized into trimethylamine N‐oxide (TMAO), which has been implicated in the progression of AS through various mechanisms.^53^, ^54^, ^55^, ^56^

## 5 CONCLUSIONS

In conclusions, the above findings hint that these metabolites may serve as novel biomarkers for early diagnosis of AS associated with genetic susceptibility. Our study identified changes similar to those observed in human and rodent models, as well as new discoveries that provide novel insights into further research on the pathophysiological mechanisms and early diagnosis of AS. However, the number of animals in this study is insufficient, highlighting the need for further verification and in‐depth exploration in further research.

## AUTHOR CONTRIBUTIONS

Hua Chen and Yunxiao Jia designed and directed the project, analyzed the data, and wrote the manuscript. Yunxiao Jia, Yuqiong Zhao, Miaomiao Niu, Changqi Zhao, and Xuezhuang Li performed the experiments. Hua Chen is the guarantor of this work and takes responsibility for the integrity of the data and the accuracy of the data analysis.

## FUNDING INFORMATION

This study was supported by the Special Scientific Research Project of Laboratory Animals (numbers: SYDW[2018]14, SYDW[2020]01, and SYDW‐KY[2021]03).

## CONFLICT OF INTEREST STATEMENT

All authors read and approved the final manuscript. The authors declare that they have no known competing interests, and there is no conflict of interest with any financial organization regarding the material discussed in the manuscript.

## ETHICS STATEMENT

All experiments involving animals were approved by the Institutional Animal Care and Use Committee of Chinese PLA General Hospital (approval number: 2018‐D14‐26). During the implementation, we strictly followed by the committee's ethical guiding principles of animal welfare.

## Supporting information


**Figure S1.** Representative histological images of abdominal aortic lesions stained with hematoxylin and eosin (H&E) (100×). (A) The plaque lesion in the atherosclerosis susceptible group (SA) mainly consisted of fibrous plaque with numerous proliferating smooth muscle cells, as well as infiltrating foam cells and inflammatory cells (b). (B) The lesion in the atherosclerosis non‐susceptible group (NSA) primarily exhibited fatty streak lesions and mild smooth muscle hyperplasia without obvious fibrous plaques (c). (a) The tunica media of abdominal aortic.

**Figure S2.** Volcanic maps of the fold change (FC) analysis between atherosclerosis susceptible group (SA) and atherosclerosis non‐susceptible group (NSA) from 0 to 7 months. (A) Negative ion modes. (B) Positive ion modes. Red represents differential metabolites with FC > 1.5 and *p* < 0.05. Blue represents differential metabolites with FC < 0.67 and *p* < 0.05.

**Figure S3.** Results of the dynamic analysis of 39 metabolites during 0–8 months (*p < 0.05; **p < 0.01).
