# Supplementary figures and images for: Preliminary study of metabonomic changes during the progression of atherosclerosis in miniature pigs
Source: Animal Model Exp Med. 2024 Jun 25;7(4):419–32. doi: 10.1002/ame2.12462 (PMC11369038; doi:10.1002/ame2.12462)

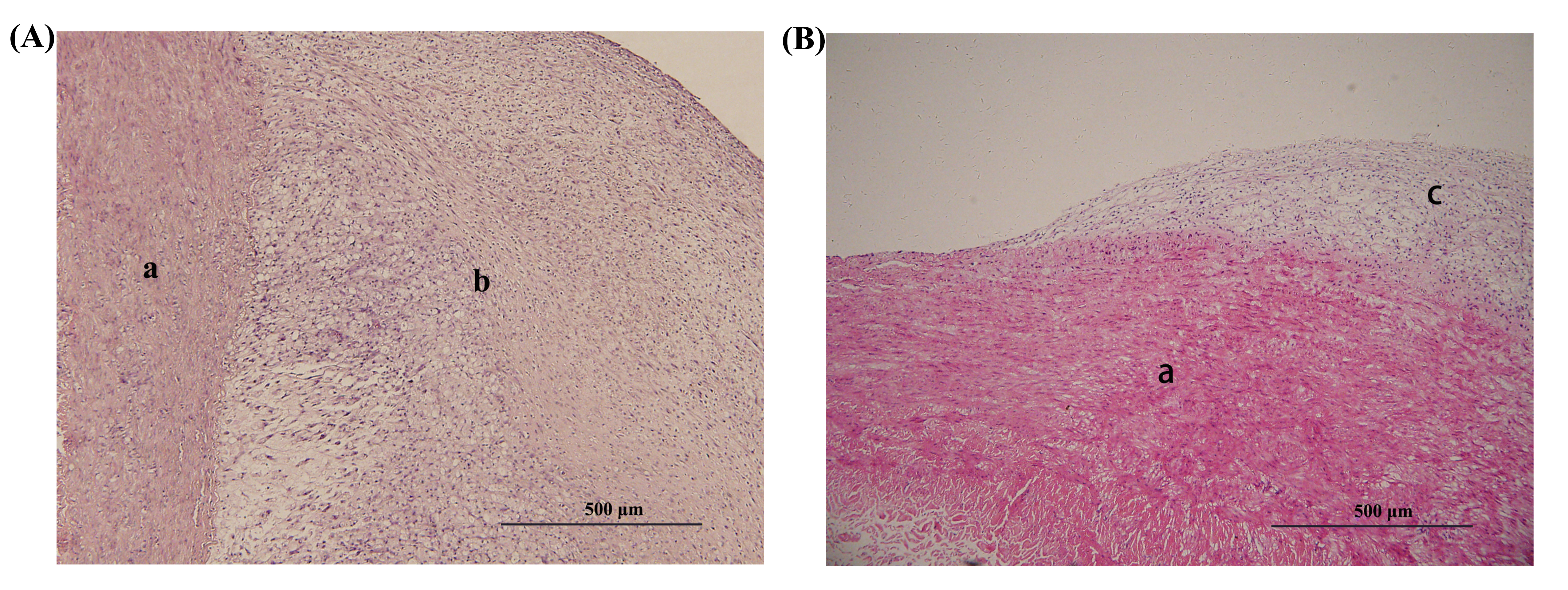

Supplement: Supplementary file 1 — Figure S1. Representative histological images of abdominal aortic lesions stained with hematoxylin and eosin (H&E) (100×). (A) The plaque lesion in the atherosclerosis susceptible group (SA) mainly consisted of fibrous plaque with numerous proliferating smooth muscle cells, as well as infiltrating foam cells and inflammatory cells (b). (B) The lesion in the atherosclerosis non‐susceptible group (NSA) primarily exhibited fatty streak lesions and mild smooth muscle hyperplasia without obvious fibrous plaques (c). (a) The tunica media of abdominal aortic. [file AME2-7-419-s001.jpg]

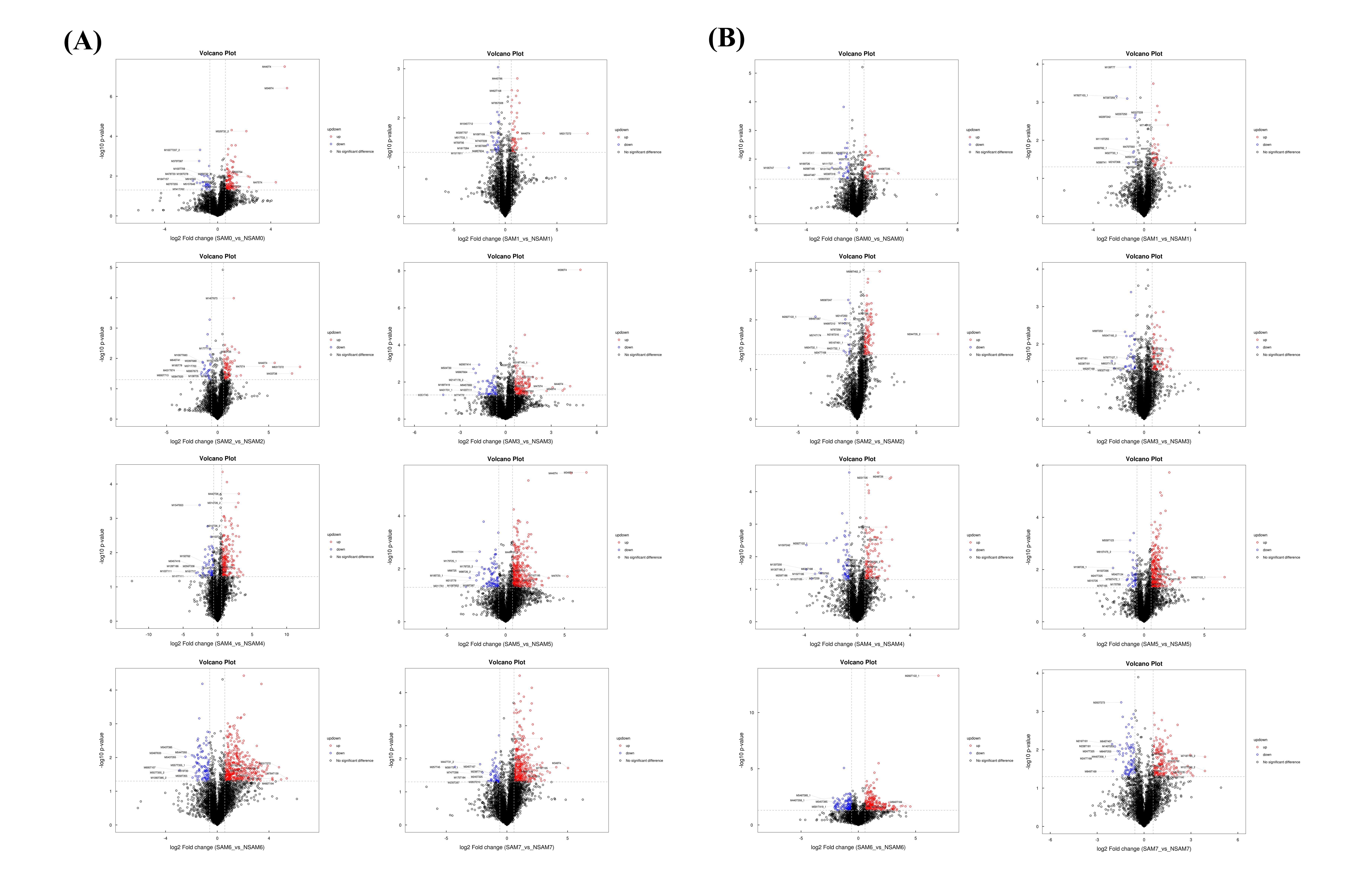

Supplement: Supplementary file 2 — Figure S2. Volcanic maps of the fold change (FC) analysis between atherosclerosis susceptible group (SA) and atherosclerosis non‐susceptible group (NSA) from 0 to 7 months. (A) Negative ion modes. (B) Positive ion modes. Red represents differential metabolites with FC > 1.5 and p < 0.05. Blue represents differential metabolites with FC < 0.67 and p < 0.05. [file AME2-7-419-s003.jpg]

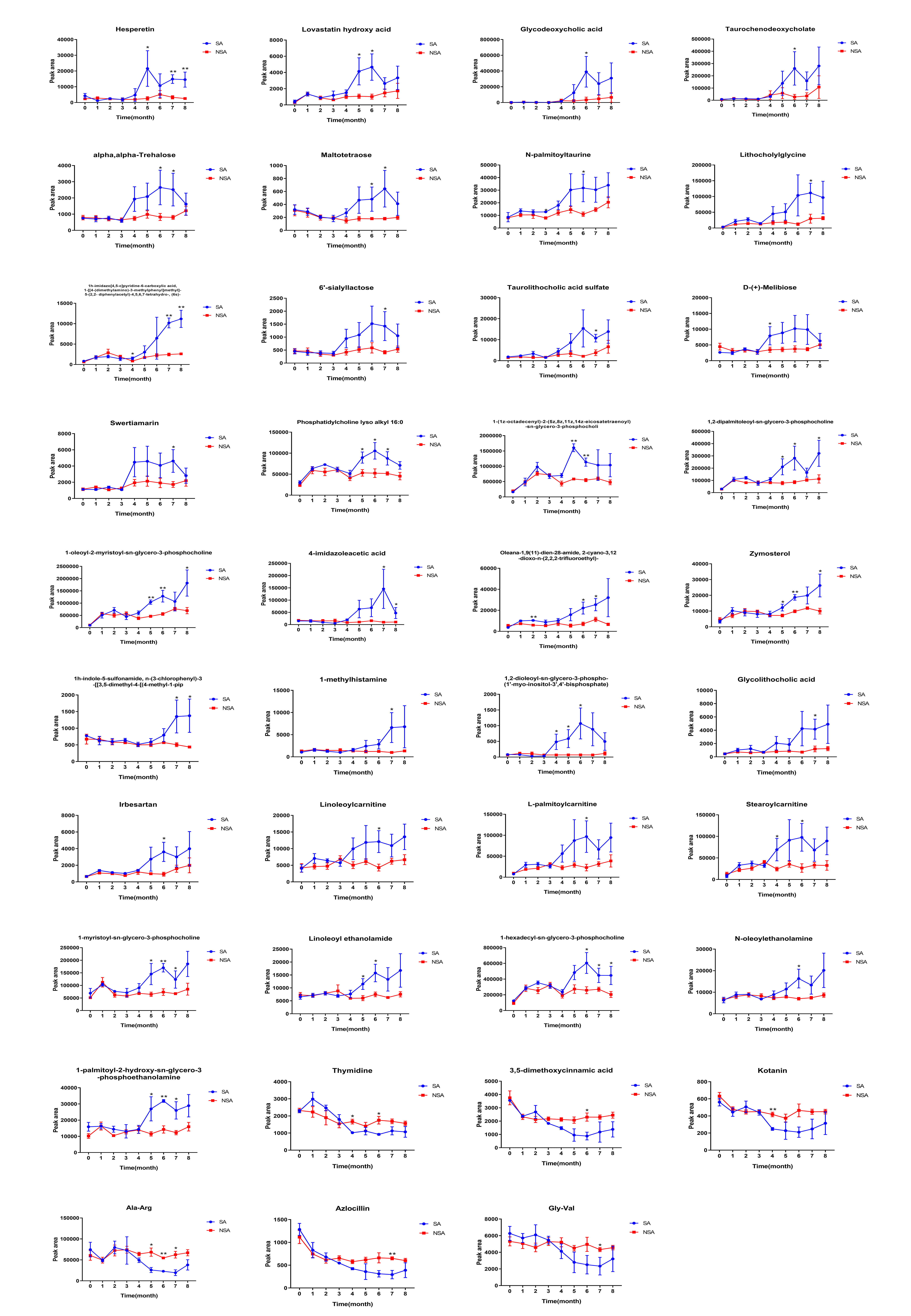

Supplement: Supplementary file 3 — Figure S3. Results of the dynamic analysis of 39 metabolites during 0–8 months (*p < 0.05; **p < 0.01). [file AME2-7-419-s002.jpg]
